# ADVANCED ILLNESS AND THE AGING NETWORK

**DOI:** 10.1093/geroni/igz038.857

**Published:** 2019-11-08

**Authors:** Andrew MacPherson

**Affiliations:** Coalition to End Social Isolation & Loneliness, Washington, District of Columbia, United States

## Abstract

This session will provide insights on the many ways that the aging network and the Older Americans Act is able to assist individuals, and their families, who are dealing with a serious or advanced illness.

